# Accounting for environmental and observer effects in estimating abundance of southern bluefin tuna from aerial survey data

**DOI:** 10.1371/journal.pone.0207790

**Published:** 2018-11-26

**Authors:** J. Paige Eveson, Mark V. Bravington, Jessica H. Farley

**Affiliations:** 1 Oceans and Atmosphere, CSIRO, Hobart, Tasmania, Australia; 2 Data 61, CSIRO, Hobart, Tasmania, Australia; Technical University of Denmark, DENMARK

## Abstract

Southern bluefin tuna (SBT) is a valuable species that has been subject to high exploitation rates since the 1950s. In 2011, the spawning stock biomass was estimated to be at a historically low level, at only 5% of pre-fished biomass. A key component for managing and rebuilding the stock is having reliable, fishery-independent estimates of juvenile abundance. This paper describes how such estimates have been constructed from aerial surveys of juvenile (age 2–4) SBT conducted annually in the Great Australian Bight from 1993–2000 and 2005–2009. During these surveys, observers flew along pre-set transect lines searching for surface schools of SBT. Data were collected on the location and biomass of SBT sightings, and on the environmental conditions present during the survey. Sea surface temperature (SST) was found to correlate with the size (biomass) of schools, and several environmental variables, SST and wind speed in particular, were found to correlate with the number of sightings (presumably by affecting the ability of observers to see surface schools as well as whether fish were present at the surface). In addition, observers changed over time and differed in their aptitude for spotting tuna. Thus, generalized linear mixed models (GLMMs) were used to standardize the sightings and biomass data to a common set of observers and environmental conditions in order to produce an annual time series of relative abundance estimates. These estimates, which form one of two key inputs to the management procedure used by the international Commission for the Conservation of Southern Bluefin Tuna to set the global catch quota, suggest juvenile abundance was highest in the first years of the survey (1993–1996), after which it declined and fluctuated around a level about four times lower.

## Introduction

Southern bluefin tuna (SBT) (*Thunnus maccoyii*) is a valuable commercially-fished species that has been subject to high exploitation rates since the 1950s. The species was listed as critically endangered on the IUCN Red List in 2011 [1], and while the most recent stock assessment in 2016 estimated the stock to be rebuilding (spawning stock biomass ~13% of original unfished biomass, an improvement from the historically low level of ~5% in 2011), it remains at a low state [2]. Recruitment, defined here as the abundance of juveniles entering the harvestable population, is one of the fundamental quantities influencing estimates of stock status and likely outcomes of different management options (e.g. [3]). Fishery-based indices of juvenile abundance derived from catch and effort data are prone to many biases, and this is particularly true for purse seine fisheries [4], which is the predominant fishery for juvenile SBT. Thus, having a reliable, fishery-independent time series of juvenile abundance estimates is key for managing and rebuilding the SBT stock. This paper describes how such a time series has been constructed from annual aerial surveys of juvenile SBT.

The aerial survey time-series is not only an important indicator for monitoring and assessing the juvenile population, but also plays a direct role in the management of SBT. As part of the rebuilding process for SBT, a management procedure—a predefined rule used to determine the catch level required to meet a specific rebuilding target [5]—was recently adopted by the Commission for the Conservation of Southern Bluefin Tuna [6]. The aerial survey estimates form one of two key data inputs to the management procedure (the other being longline catch-per-unit-effort data on sub-adult and adult fish), and are the only fishery-independent input [7].

Aerial and ship-based line-transect surveys are commonly used for estimating abundance of terrestrial and marine animals (see [8]), with distance sampling being the customary method of analysis (e.g., [9] and references therein). However, aerial sightings data for SBT have some properties which mean they are not amenable to distance sampling methods. Most notably, the definition of a sighting and its distance from the transect line is imprecise for SBT since an aggregation of multiple schools may be deemed one or more sightings, depending on its location relative to the transect line and on the judgement of the observer (see Field Procedures section). As such, we apply something more akin to a strip-transect analysis where there is no need for an accurate measurement of distance from the line because all schools within the strip are assumed to be equally detectable.

Environmental variables such as sea surface temperature and wind speed have strong effects on the amount of tuna spotted (by affecting both their presence at the surface and how easily they can be detected) and may also affect the schooling structure of tuna. Furthermore, observers operating in the survey vary in their aptitude for spotting tuna. Since both environmental variables and observers can differ from year to year, it is important to standardize for these covariates when modelling the sightings data. To facilitate standardization, we use two separate models to predict (i) size (biomass) of a sighting and (ii) density of sightings at standardized conditions within predefined space and time strata. The two standardized predictions are combined multiplicatively, and then summed across all strata to give a relative index of biomass in each survey year. The word ‘relative’ is important here as our goal is to provide an index that indicates whether the juvenile population is increasing or decreasing, not to provide absolute abundance estimates. Although the two models can, individually, be fitted with standard software, combining the results requires more specialised methods to ensure the uncertainties within each year are propagated correctly.

## Material and methods

### Southern bluefin tuna in the Great Australian Bight

Large numbers of juvenile SBT, predominantly ages 2 to 4 years, are found in the warm continental shelf waters of the Great Australian Bight (GAB) during the austral summer [10]. Extensive electronic tagging of juveniles has shown that individuals make annual cyclic migrations between the GAB in summer and deep oceanic waters spanning from South Africa to New Zealand in winter (e.g., [11, 12]). The timing of these migrations varies between individuals but, for the most part, juveniles enter the GAB between November and January and leave the GAB between April and June. These cyclic migrations appear to stop as they get older, with relatively few fish above age 5 found in the GAB [13].

While in the GAB, SBT form surface schools which are targeted by a large-scale purse seine fishery operating from December through April. These surface schools produce a distinct ripple on the water’s surface which can be seen from the air (over 10 miles away in ideal conditions). As such, commercial aircraft with professional tuna spotters are employed by the fishing industry to help locate fish.

The concentration of juvenile SBT within a defined area and time period, their surface-orientated behaviour while in this region, and the availability of skilled tuna spotters all provide favourable conditions for conducting a scientific aerial survey.

### Field procedures

A scientific aerial survey for SBT has been carried out annually in the GAB from 1993 to 2000 and from 2005 to 2017, with the hiatus due to logistic problems finding trained observers available to participate. The survey takes place over three months, from January through March, each year. A key assumption of the survey is that (roughly) the same proportion of the total juvenile population is present each year in the GAB during survey months—this seems to be a reasonable assumption based on electronic tagging results that showed almost 100% of tagged juveniles migrated to the GAB each summer [12].

**Fig 1:**
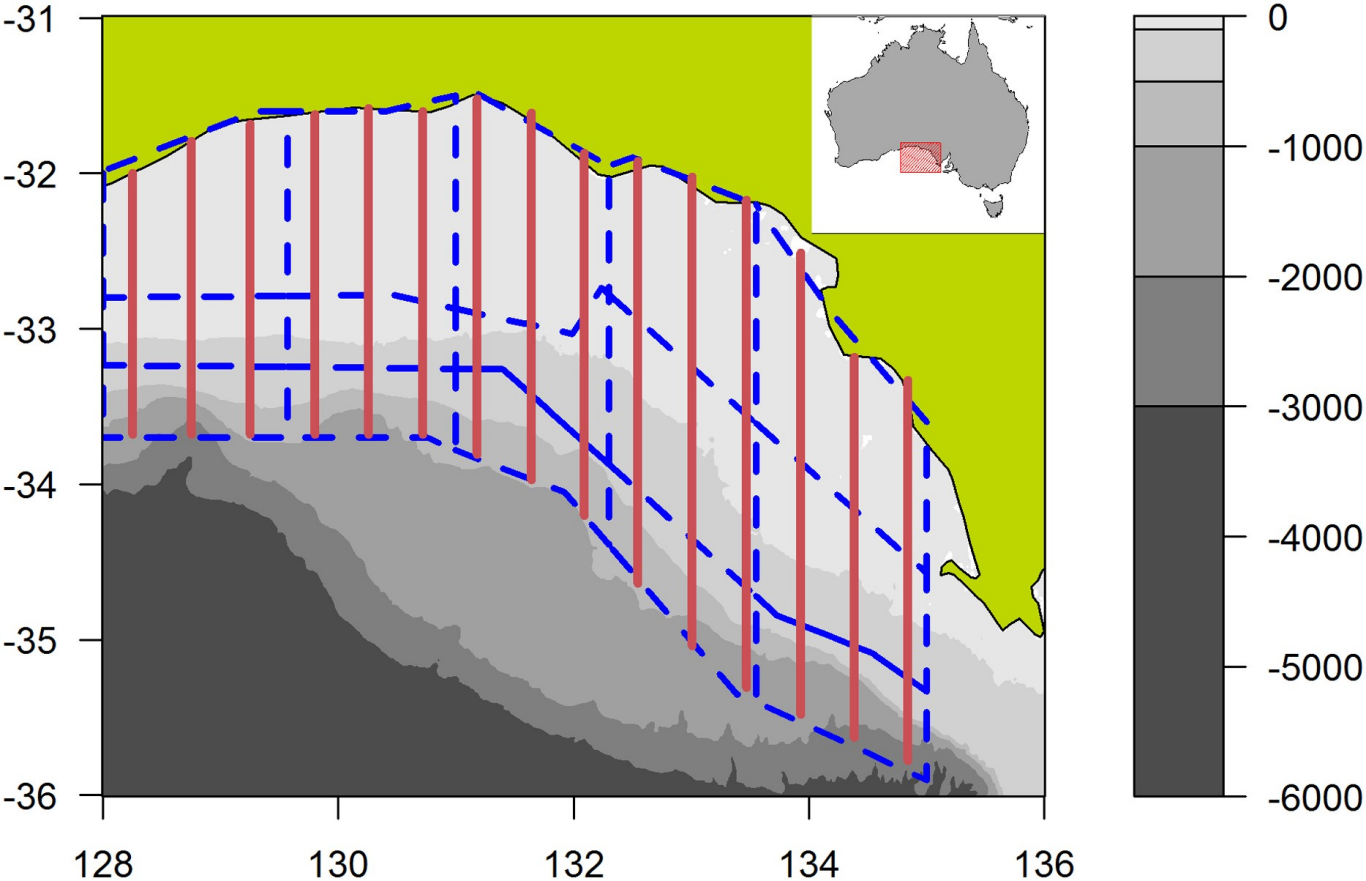
Map of aerial survey area for southern bluefin tuna in the Great Australian Bight. Solid red lines show the 15 north-south transect lines, and dashed blue lines show the 15 areas defined for analysis purposes. The depth profile (in metres) is shown in greyscale.

One or two planes (Rockwell Aero Commander 500S, with wings above the windows) have flown in each survey, depending on budget and availability (Table 1). Fifteen approximately equally spaced north-south transect lines, lying between 128°E and 135°E and running from the coast to the continental shelf, are searched for schools of SBT (Fig 1). A complete replicate of the GAB consists of a subset of 12 out of the 15 lines, excluding in rotation either lines 1, 3 and 14 or lines 2, 13 and 15, since SBT abundance has been historically low in the easternmost and westernmost regions of the survey area. To the greatest extent possible, a complete replicate is surveyed before starting another replicate.

**Table 1:** Spotter pairs by plane and survey year.

Year	Plane 1			Plane 2	
1993	5, 7	5, 4	5, 8		
1994	5, 7	5, 4		8, 7	8, 4
1995	5, 7	5, 4	5, 3	8, 7	8, 4
1996	5, 7	5, 3		4, 7	4, 3
1997	5, 7	5, 3		4, 7	4, 3
1998	5, 7	5, 3		4, 7	4, 3
1999	10, 6	10, 9		4, 6	4, 9
2000	10, 1	10, 2		4, 1	4, 2
2005	4, 11				
2006	4, 11				
2007	4, 11	4, 3			
2008	5, 11	5, 3			
2009	5, 11	5, 3			

Numeric codes are used to maintain anonymity. The first number in each pair denotes the spotting pilot and the second denotes the dedicated spotter.

Up to and including 2009, all survey planes had two observers, also referred to as spotters—a spotting pilot and a dedicated spotter—searching simultaneously for schools of SBT (Table 1). Due to the retirement of spotting pilots and the impossibility to replace them, planes flying in the 2010 and subsequent surveys had only one spotter (along with a non-spotting pilot). Solo spotters tend to make fewer sightings than two spotters [14]. While methods to estimate and account for differences between one and two spotters have been developed based on calibration experiments run in conjunction with the 2007–2009 aerial surveys [14], this material would be lengthy to describe and is unlikely to be of general use since it is very specific to the SBT survey and analysis. As such, only data from surveys conducted prior to 2010 are considered here.

Survey protocols specify that the plane flies at a consistent speed and altitude (120 knots, 1500 ft), and a GPS is used to log waypoints. Each of the two spotters searches the sea surface from straight ahead through to 90° on his side of the plane (abeam of the plane). When a sighting is made (noting it can consist of multiple schools—see next paragraph), the plane continues along the transect line until perpendicular to the sighting, at which point the plane leaves the line and flies out to the sighting. If the spotters confirm the sighting to be SBT, the plane circles each school so that the two spotters can make independent estimates of its biomass (in tonnes). The midpoint of the sighting is recorded, noting this can be quite approximate due to the variable nature of a sighting and the constraints of circling in a plane. The plane then flies back to the point it left the transect line to resume searching.

**Fig 2:**
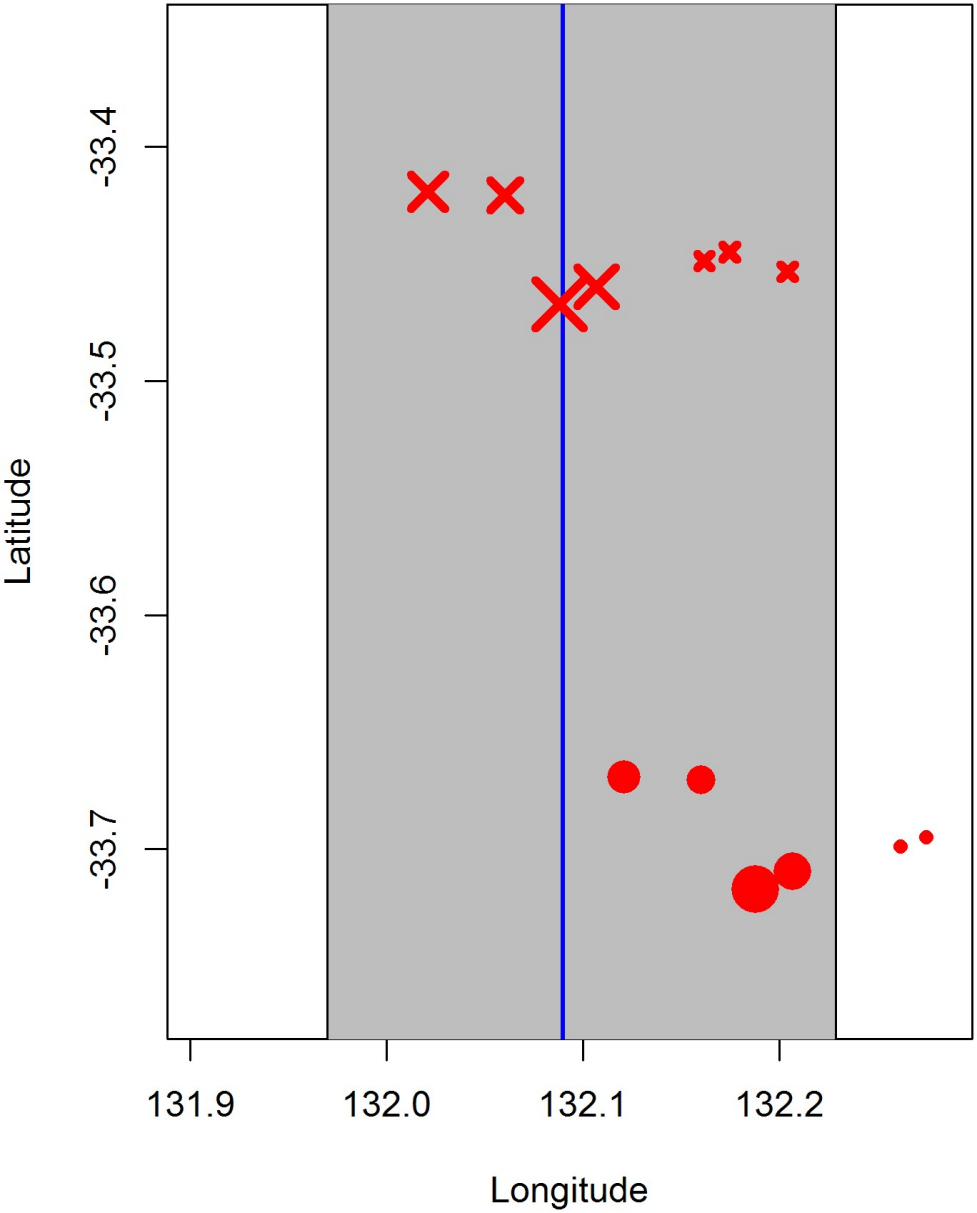
Illustration of a sighting consisting of multiple schools. The solid blue vertical line is the transect line, the grey shaded area is within ±6 nm of the transect line. The solid red circles mark an actual sighting consisting of seven schools, where the size of each circle is proportional to school tonnage (note that the three small schools outside the 6 nm strip are included in our analysis since part of the sighting was within the strip). If the location of these schools relative to the transect line had been shifted, as indicated by the red crosses, this would have been deemed at least two sightings (since spotters search their own side of the plane).

It is difficult to precisely define what constitutes a sighting for SBT. Although fish aggregate in schools, the schools themselves are often clustered and can be somewhat ephemeral and fluid. A sighting can consist of one or multiple schools (the maximum being 76 in the surveys to date), each ranging in size from less than one to over a hundred tonnes, and the schools can be tightly clustered or spread over several miles. Whether a cluster of schools is deemed to be one or more sightings can be influenced by its location relative to the transect line; for example, a cluster of schools spanning the transect line will more likely be judged multiple sightings (since each spotter searches only his side of the line) than a cluster of schools distant from the line (Fig 2). This makes the data less amenable to distance sampling methods, and motivated our use of an alternative approach.

Although efforts have been made to use the same spotters each year, or at least to have overlap between spotters and years, this has not always been possible (Table 1). For example, in 1999 and 2000, a shortage of experienced spotters meant that the spotting pilot in one of the planes (code 10) was new to the survey (albeit experienced in aerial spotting for the commercial SBT fishery) and the dedicated spotters in both planes were trainees (codes 1, 2, 6 and 9). The trainee spotters did not estimate the biomass of schools, so for sightings made during flights with trainees, only biomass estimates made by the spotting pilot are available.

Environmental observations are recorded at the start and end of each transect line and at approximately 30-minute intervals during the transect flight. The variables recorded include wind speed (knots) and direction, air temperature (°C), amount of high cloud or ‘sea shadow’ (0 to 8 octas), and level of haze and swell (both on a scale of 0 to 3—none, slight, moderate, severe—as judged by the observers). Sea surface temperature (SST, °C) is later extracted from remote sensing data; we use a 3-day composite satellite SST dataset produced for the Australasian region [15].

The survey is only conducted when the environmental conditions are suitable (i.e., wind speed <10 knots and visibility good out to 7 nautical miles). Therefore, even though the survey design is balanced in space and time, the weather does not always permit full replicates to be completed.

### Model overview

We modelled the data using a strip-transect approach, in which all sightings made within 6 nautical miles (nm) on either side of the transect line were included in the analysis and treated as equally probable. This approach was deemed suitable because the sightings data showed no reduction in the biomass or number of schools of SBT spotted out to 6 nm from the transect line (see Results). The distance between transect lines is ~24 nm, so using 12 nm strips covers approximately half of the survey area (Fig 1). When a sighting consisted of more than one school, it was included in the analysis if at least one of the schools was within 6 nm of the line (e.g., Fig 2). For modelling purposes, we divided each of the five longitudinal strips into three sections, where the latitudinal divisions were chosen to correspond roughly to depth strata (inshore, mid-shore and shelf-break), resulting in 15 areas (Fig 1).

Separate models were constructed to describe two different components of observed biomass: i) biomass per sighting (BpS), and ii) sightings per mile of transect line (SpM). This allowed the effect of environmental and observer covariates on the two components to be modelled differently, as appropriate. For example, we would expect the level of haze to have a greater effect on the number of sightings (SpM) due to reduced visibility than on the size of a sighting (BpS). Each component was modelled using a generalized linear mixed model (GLMM). Year, month and area, and all possible interactions between them, were included in the models so that the observed data were adjusted primarily for environmental effects, not for space and time factors. The main effects for year, month and area were modelled as fixed effects and the 2-way and 3-way interaction terms were modelled as random effects. Using random effects allowed us to effectively deal with the fact that many of the 2-way and 3-way strata have little or no search effort (an issue for the SpM model), and few or no sightings (an issue for the BpS model).

**Fig 3:**
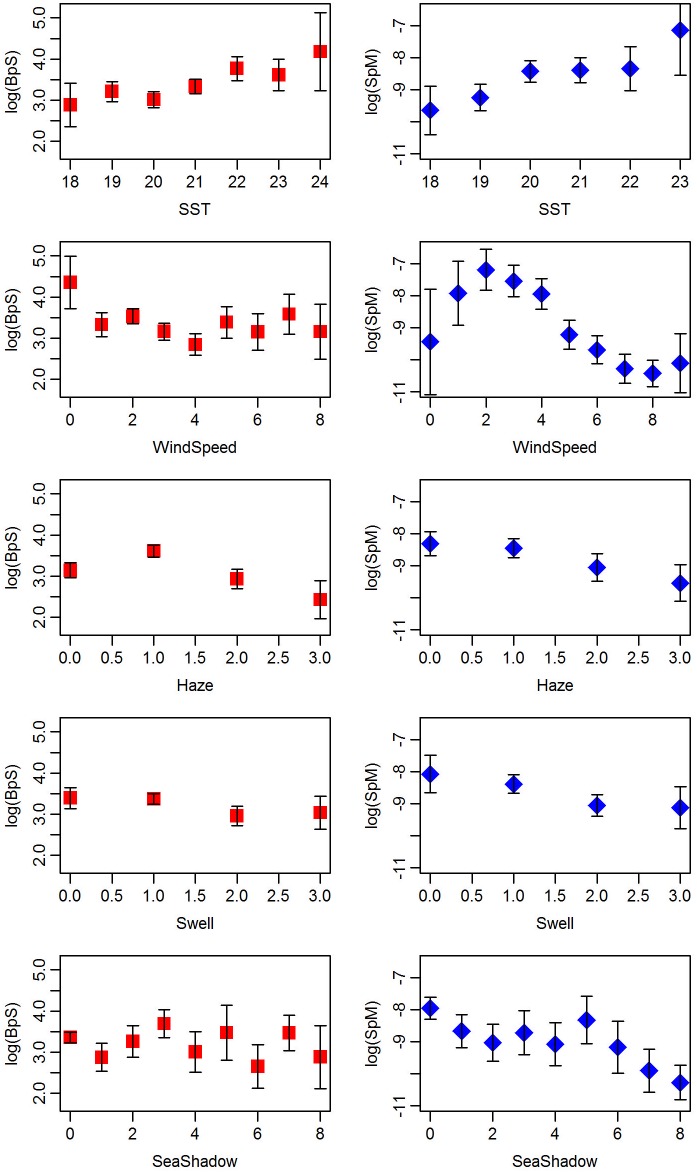
Biomass per sighting (left) and number of sightings per mile (right) (both on a log scale) versus candidate environmental variables. Shown are the means ± 2 standard error bars.

The environmental variables considered for inclusion in the models were wind speed, haze, swell, sea shadow and SST. We did not consider air temperature because it correlates highly with SST (~0.7); the correlation was low between all other covariates (ranging from 0.01–0.27 in absolute value). The environmental covariates were included in the models as linear terms because exploratory plots of the data (Fig 3) and preliminary analyses using generalized additive models suggested this was appropriate. In cases where there was some evidence of a more complex relationship (in particular, wind speed in the SpM model), this was due to curvature at the extreme ranges of the covariate where the data are sparse and uncertainty is high. To select which of the candidate environmental variables to include in the models, we began by including all variables as additive terms. We then used Wald tests of significance for each parameter estimate provided by the model-fitting software (see next paragraph), along with AIC, to determine which of the variables to retain in the final models. A problem with using AIC to compare mixed effect models is how many parameters to associate with the random effects; however, when comparing models that differ only in their fixed effects, this is not an issue. We note that we also explored models that included two-way interaction terms between environmental variables, however we found few of the interactions were supported. Furthermore, for those interactions that were found to be significant, we were concerned they could be spurious since several variables have the same value for a large percent of the observations (e.g. sea shadow is recorded as 0 almost 50% of the time, and swell is recorded as 1 over 50% of the time), so when crossed with another variable, many combinations have very few observations.

To fit the GLMMs, we used the ‘gam’ function in the mgcv package [16] developed for the R statistical computing environment [17]. Even though we did not model the environmental covariates as smooths, we used ‘gam’ because it allows for random effects to be included in the models in the same way as smooths (namely as penalized regression terms; see [16] for details), whilst also allowing for linear covariates. There are other options in R for fitting GLMMs (many of which became available after the aerial survey analysis was first developed), but we found ‘gam’ to be stable and have the functionality we required (plus it allowed us to explore the use of smooths for the environmental covariates).

To evaluate the model fits, we used the diagnostic tools provided by the DHARMa package in R [18]. Evaluating model fits for GLMMs is difficult because standard residual plots can suggest problems even if the model is correctly specified. DHARMa overcomes this by simulating residuals from the fitted GLMM that are standardized to values between 0 and 1, and can be interpreted similarly to residuals for linear models. Details can be found in the package documentation and in the vignette obtained by typing vignette("DHARMa", package = "DHARMa") in R. The package includes statistical tests of goodness of fit, overdispersion and zero inflation (see S3 Appendix).

Details of the BpS and SpM models are given in subsections below. Once these models were fitted, they were used to predict what the average biomass of a sighting and the number of sightings per 100 nm of search distance would have been in each year, month and area under standardized environmental conditions and the same set of observers. Note that the predictions are conditional (not marginal) predictions that include the random effects. We chose to standardize to average conditions and the best observer pair, noting that this choice is not important for estimating relative abundance (i.e., choosing above/below average conditions would simply shift the annual estimates up/down but would not change their value relative to each other). We then obtained an estimate of standardized abundance in each year:month:area stratum as follows:

$${\hat{A}}_{ijk}={\hat{S}}_{ijk}{\hat{B}}_{ijk}$$

where ${\hat{B}}_{ijk}$ and ${\hat{S}}_{ijk}$ denote the predicted values of BpS and SpM, respectively, in year *i*, month *j* and area *k* under standardized environmental/observer conditions. Taking an area-weighted sum over all stratum-specific abundance estimates within a year gives a standardized abundance estimate for that year, namely

$${\hat{A}}_{i}=\sum_{j}\sum_{k}w_{k}{\hat{A}}_{ijk}$$

where *w*_*k*_ is the geographical size of area *k* relative to the entire survey area;$\sum_{k}w_{k}=1$.

To ensure correct interpretation of the standardized annual abundance estimates as relative indices, we divided the annual estimates by their mean such that the final index has a mean of 1; i.e.

$${\hat{I}}_{i}=\frac{{\hat{A}}_{i}}{\frac{1}{n}\sum_{i=1}^{n}{\hat{A}}_{i}}$$

#### Biomass per sighting (BpS) model

The response variable in the BpS model is the biomass of each sighting; however, each sighting (or, more specifically, each school) has two estimates of biomass—one from each observer. Because our ultimate goal is to obtain a relative abundance index, we do not need to know which observer’s estimates are most accurate, but rather whether one observer systematically under or over estimates school size relative to another observer. Provided there is sufficient overlap between observers across years, it is possible to inter-calibrate school size estimates for all observers by fitting a generalized linear model to the differences in paired biomass estimates. One observer is chosen as a reference, and for every other observer, the model estimates a parameter representing the factor by which this observer’s biomass estimates differ, on average, from the reference observer’s estimates. Details are provided in Supporting Information (S1 Appendix).

After fitting the biomass observer model, the original school size estimates were adjusted using the estimated observer effects (i.e., school size estimates made by observer A were multiplied by the parameter estimates for observer A). Because the observer effects were estimated with high precision (see Results), we treated the adjusted school size estimates as exact in our subsequent analyses. A final biomass estimate for each school was calculated as the average of the two adjusted estimates, then the final school size estimates within each sighting were aggregated to give total biomass per sighting (BpS) estimates.

The BpS estimates were modelled using a GLMM with a Gamma error structure and a log link function. As noted above, year, month and area were included as fixed factors, and all 2-way and 3-way interactions between year, month and area were included as random effects. The only environmental variable found to have a significant effect was SST (see Results). Thus, the model for the expected biomass of sighting *l* in year *i*, month *j* and area *k* can be expressed as:

$$\text{log}\left(E\left[BpS_{ijkl}\right]\right)=\beta_{1i}+\beta_{2j}+\beta_{3k}+b_{1ij}+b_{2ik}+b_{3jk}+b_{4ijk}+\gamma \;SST_{ijkl}$$

where *β*_1_, *β*_2_ and *β*_3_ are the fixed effects for year, month and area respectively, and *b*_1_, *b*_2_, *b*_3_ and *b*_4_ are the random effects for the year:month, year:area, month:area and year:month:area interactions.

#### Sightings per mile (SpM) model

For the SpM model, we first conducted an analysis to get estimates of the relative “sighting efficiency” of all observer pairs that flew in the 1993–2009 surveys (i.e., the probability that an observer pair would not miss an available sighting). Because spotters operate in pairs, it is possible to cross-calibrate individual observers by comparing sighting rates within single flights, before having to take into account space, time, and environmental factors. The rationale is that abundance and sighting conditions are roughly the same for both observers within a flight, so within-flight differences between the number of sightings by the two observers should largely be attributable to differences in observer sighting efficiency. Details are provided in Supporting Information (S1 Appendix). After we fitted the observer pair relative efficiency model, the estimates were included in the SpM model as an offset with known coefficient of one (see below).

We could not simply include observer in the model as a categorical covariate (where observer would be the spotter who made the sighting) because the same spotters did not operate in all years, so it would not be possible to estimate a fixed effect for each of them. One possible solution might be to include observer as a random effect term (as was done in [19]), except there are reasons why this is not appropriate in our situation. The standard protocol for double-observer line-transect surveys is for each observer to remain unaware of what the other has seen. However, in the cramped confines of the planes in the SBT aerial survey, this is not possible, so the sightings made by the two observers are not independent. For these reasons we needed to develop alternative methods.

The data for the SpM model were accumulated by flight (which is unique to a given year, month, day and plane) and area for every flight-area combination in which search effort was made (even if no sightings were made). Within each flight-area combination, the number of sightings and the distance flown were summed, whereas the environmental conditions were averaged. The number of sightings was used as the response variable in the GLMM, as opposed to the sightings rate; the model was then fitted assuming a Tweedie error structure with a log link and including distance flown (on a log scale) as an offset term. We also tried using a Poisson distribution but it has a very strict variance structure in which the variance is equal to the mean, and it underestimated the amount of variance as well as the number of zero observations (see S3 Appendix). The Tweedie distribution has variance equal to *φμ*^*p*^, where *μ* is the mean, *φ* is a scale (overdispersion) parameter, and *p* is a power parameter. A value of *p* = 1 corresponds to a Poisson distribution, *p* = 2 corresponds to a gamma distribution, and 1< *p* <2 corresponds to a compound Poisson–gamma distribution with a positive mass at zero.

Year, month and area were included as fixed factors, and all 2-way and 3-way interactions between year, month and area were included as random effects. All of the environmental covariates considered were found to significantly affect the number of sightings, so SST, wind speed, haze, swell and sea shadow were included in the model as linear covariates (see Results). Thus, the model for the expected number of sightings made in year *i*, month *j* and area *k* during flight *t* can be expressed as:

$$\begin{array}{l} \text{log}\left(E\left[SpM_{ijkt}\right]\right)=\text{log}\left(Dist_{ijkt}\right)+\text{log}\left(Obs_{t}\right)+\beta_{1i}+\beta_{2j}+\beta_{3k}+b_{1ij}+b_{2ik}+b_{3jk}+b_{4ijk}+\\ \;\gamma_{1}\;SST_{ijkt}+\gamma_{2}\;Wind_{ijkt}+\gamma_{3}\;Haze_{ijkt}+\gamma_{4}\;Swell_{ijkt}+\gamma_{5}\;Shadow_{ijkt} \end{array}$$

where *β*_1_, *β*_2_ and *β*_3_ are the fixed effects for year, month and area respectively, and *b*_1_, *b*_2_, *b*_3_ and *b*_4_ are the random effects for the year:month, year:area, month:area and year:month:area interactions; and log(*Dist*) and log(*Obs*) are the offset terms for distance flown and observer pair efficiency. By including observer pair efficiency as an additive offset term on the log scale, the number of sightings effectively gets scaled by the estimated relative efficiency of the observer pair who made the sightings. We acknowledge that this approach does not account for uncertainty in the observer pair efficiency estimates; see Discussion.

### Estimating uncertainty in abundance estimates

To estimate uncertainty in the standardized annual abundance estimates, we need to incorporate both sampling variability (controlled by the sample size, which in our case is the amount of search effort) and parameter uncertainty (i.e., uncertainty in the estimates of the environmental covariates, and ideally in the observer pair efficiency estimates as well, but as noted above and in the Discussion, this is not done here). The latter source of uncertainty means the stratum-specific standardized abundance estimates are not independent, because any error in the estimated effect of, say, SST will affect all strata equally. Thus, calculating the variance of the sum of the stratum-specific abundance estimates becomes more complicated. To deal with this, we first calculated the variance of the the stratum-specific estimates conditional on the estimated environmental covariates. These conditional estimates should be independent, so we could calculate the variance of their (area-weighted) sum within each year using standard statistical principles. We then needed to calculate the additional variance due to uncertainty in the environmental covariates; this was done by calculating the numerical derivative of the standardized annual abundance estimates with respect to each of the environmental covariates and applying the chain rule. The sum of the conditional variance and this additional environmental variance gives the total variance of the standardized annual abundance estimates. Details of all steps can be found in Supporting Information (S2 Appendix).

Coefficients of variation for the standardized annual abundance estimates were calculated by dividing the square root of the variance estimates by the abundance estimates themselves. To calculate confidence intervals, we assumed that the log-transformed estimates follow a normal distribution with standard errors approximated by the coefficients of variation of the untransformed estimates. That is, an approximate 95% confidence interval for the standardized abundance estimate in year *i* (*A*_*i*_) was calculated as: $\text{exp}\left(\text{log}(A_{i})\pm 1.96\sqrt{Var(A_{i})}/A_{i}\right)$.

## Results

Total search effort (i.e., distance flown along transect lines) varied greatly between survey years depending on availability of planes and on weather conditions (Table 2). The number of sightings per 100 nm of transect line and the average biomass of sightings also varied significantly (Table 2), but do not appear to be correlated.

**Table 2:** Summary of aerial survey data by year.

Survey year	Total distance searched (nm)	Number sightings	Number sightings per 100 nm	Mean biomass (t) per sighting	Max biomass (t) per sighting	Mean schools per sighting	Max schools per sighting
1993	7603	129	1.70	94.5	1426	3.9	76
1994	15180	160	1.05	87.2	631	3.3	23
1995	14573	165	1.13	121.0	1389	3.5	38
1996	12284	110	0.90	144.4	1763	4.0	46
1997	8813	101	1.15	89.5	852	3.2	18
1998	8550	104	1.22	92.6	1895	2.2	21
1999	7555	50	0.66	59.5	993	2.5	21
2000	6775	76	1.12	62.6	414	2.6	17
2005	5968	79	1.32	74.1	398	2.4	17
2006	5150	43	0.83	91.4	958	2.0	8
2007	4872	41	0.84	84.0	410	2.6	11
2008	7462	121	1.62	65.6	614	3.5	24
2009	8101	145	1.79	53.9	896	2.5	22

All biomass statistics have been adjusted for relative differences in school size estimates between observers.

**Fig 4:**
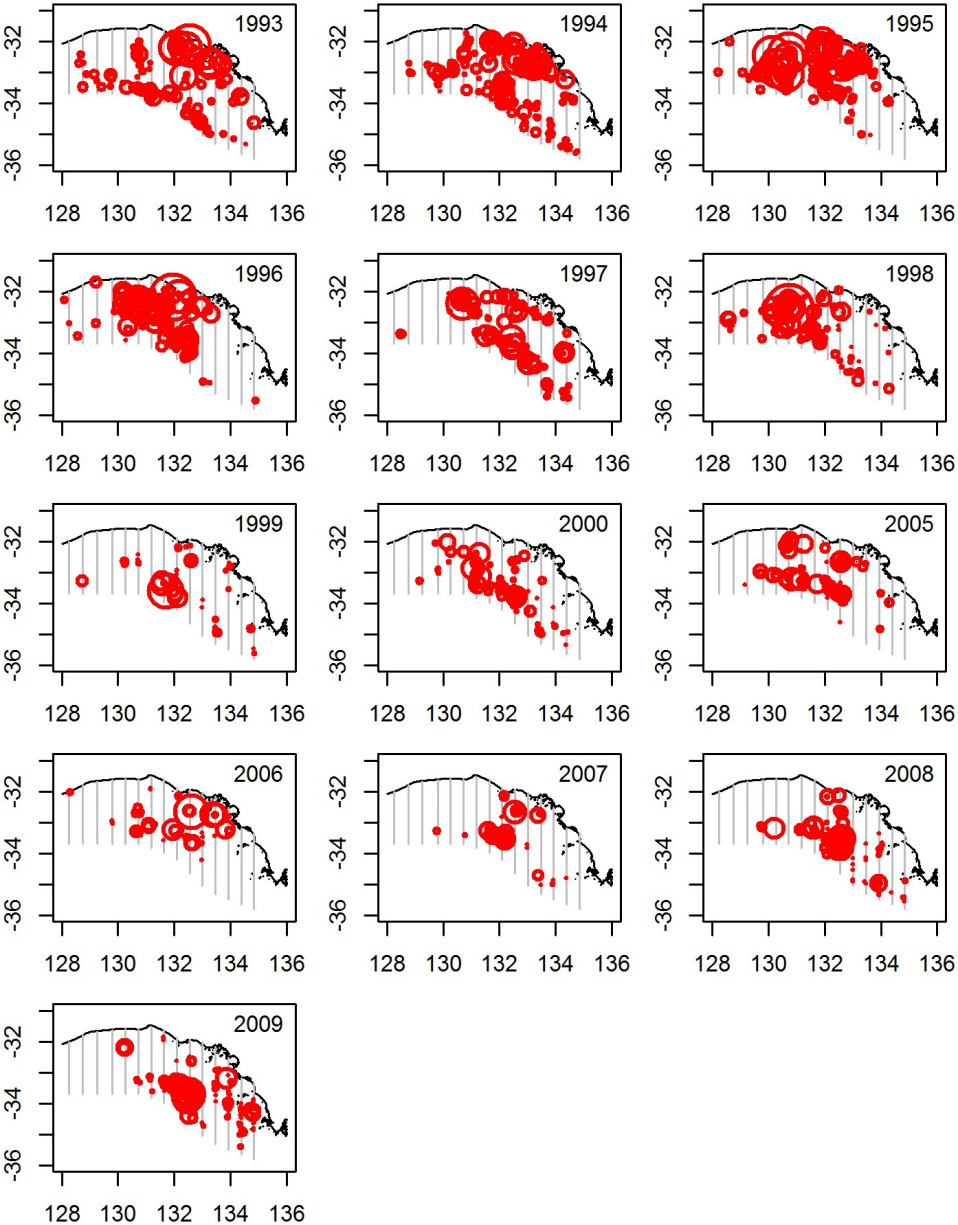
Distribution of sightings made during each survey year. Red circles show the locations of sightings, where the size of the circle is proportional to the square root of the sighting biomass. Grey lines show the 15 transect lines.

Sightings tend to be distributed inshore and along the shelf break, although this pattern is more evident in some years than others (Fig 4). There were almost no sightings in the westernmost block (lines 1 to 3) in any years, and the number of sightings in the easternmost block (lines 12 to 15) tended to be low, with 2009 being an exception.

**Fig 5:**
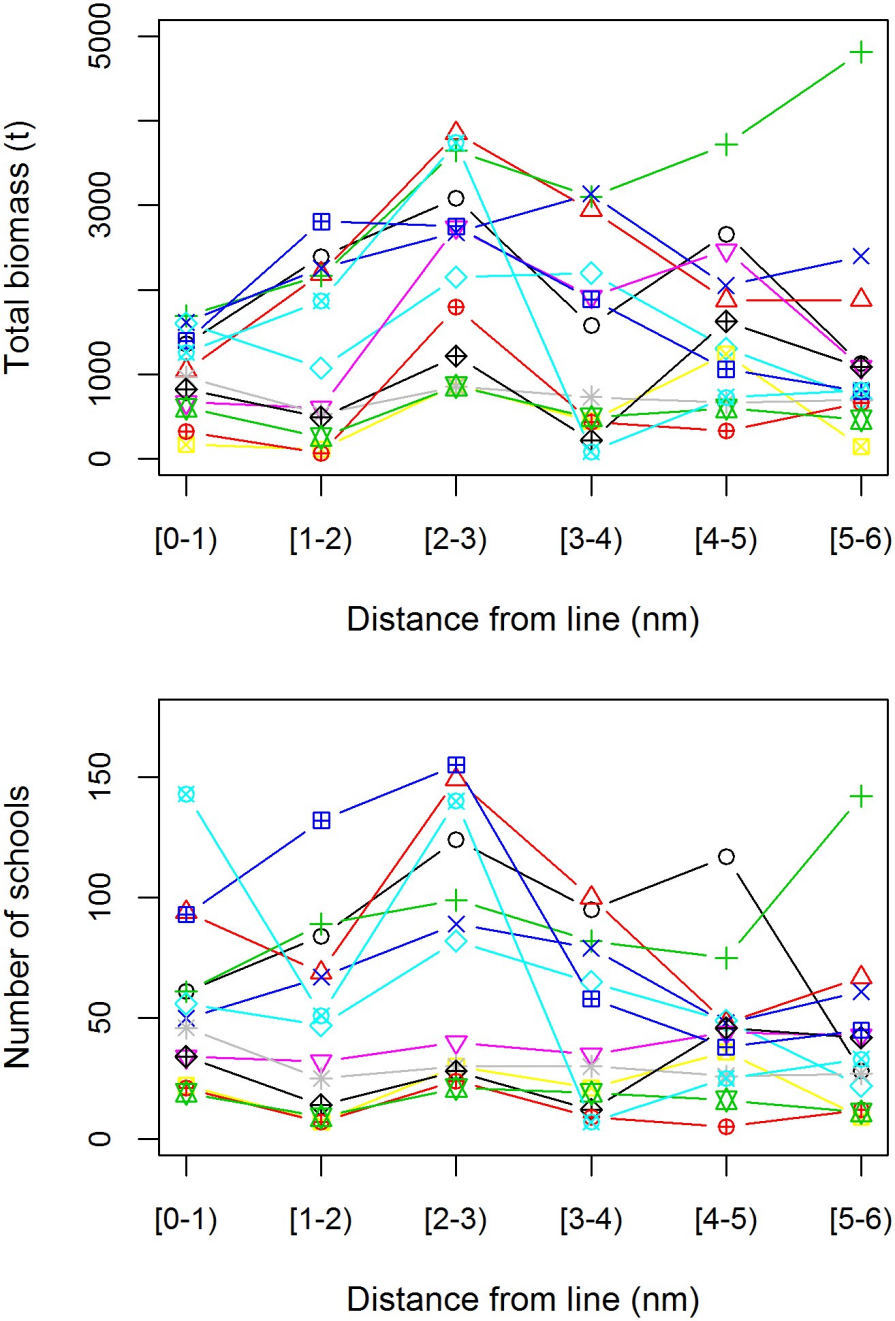
Total biomass of sightings (top) and number of schools sighted (bottom) versus distance of sightings from the transect line. Each symbol and colour represents a different survey year.

Total biomass of sightings in a year remained relatively constant with distance from the transect line, as did the number of schools sighted (Fig 5), with the exception of a small peak in both around 2–3 nm from the line. The reason for such a peak is unknown, and would be an issue if a distance sampling approach had been used (since it suggests a detection function that does not equal 1 on the line and is not monotonically decreasing). However, with the strip transect approach we are using, it should not bias a relative abundance index. Interestingly, although the number of schools was reasonably constant with distance from the line, the number of sightings declined and the average number of schools per sighting increased, supporting our earlier hypothesis that spotters will have a greater tendency to group schools that are further from the transect line into a single sighting.

**Fig 6:**
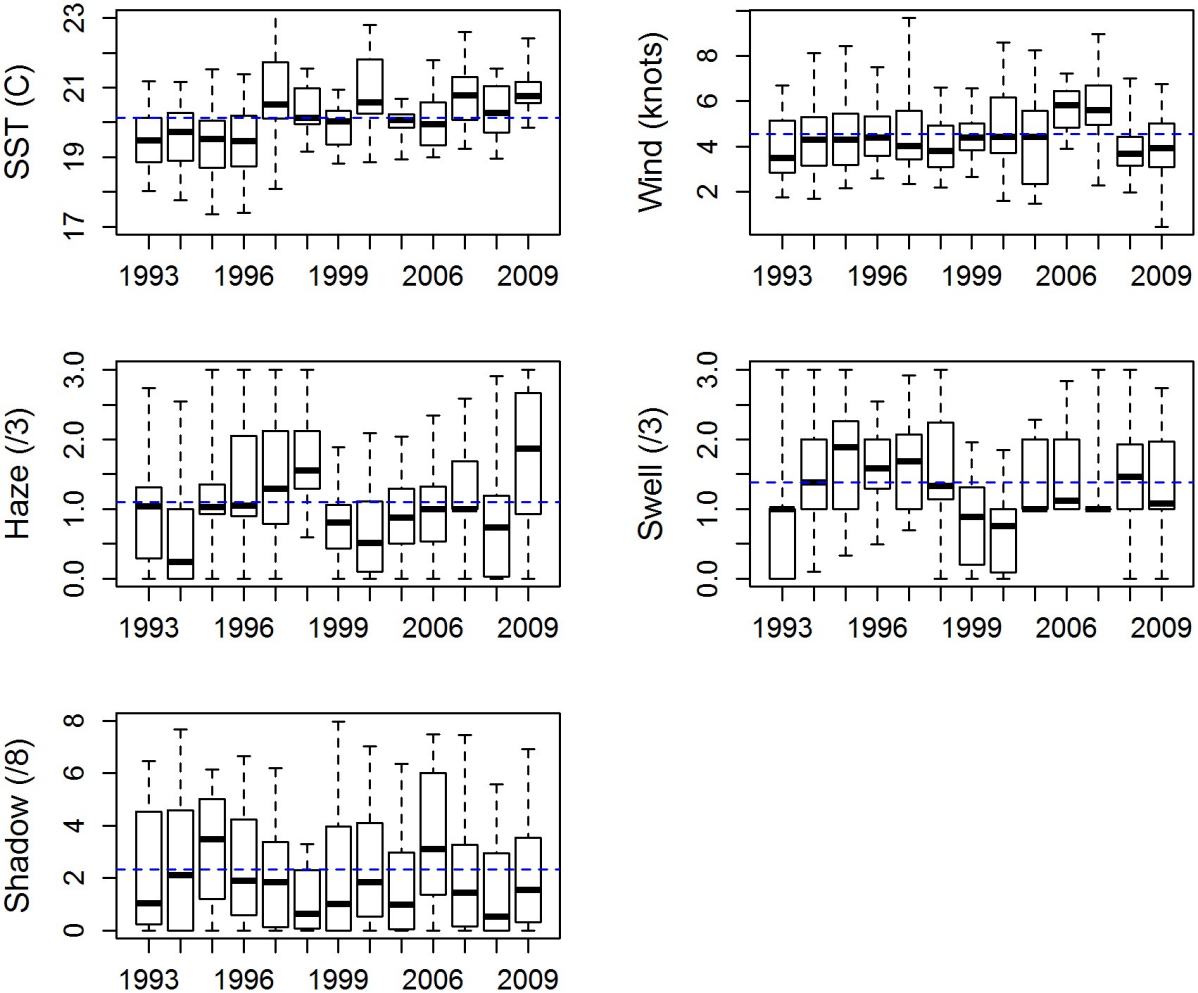
Box-and-whisker plots summarizing the environmental conditions present during search effort for each survey year. The boxes show the median and the inter-quartile range, and the whiskers extend to the minimum and maximum values. The dashed blue horizontal line across each plot shows the average across all survey years.

The environmental conditions that were present during search effort varied substantially among years for most variables, with an overall increasing trend in SST (Fig 6). The trend in SST over time would be an issue if it was confounded with a year effect; however, the positive relationship between SST and sightings (as seen in Fig 3) is apparent even within years, so the model should be able to separate these effects. Within a given survey year, different environmental variables often act in opposite directions for standardization purposes (e.g., in 1993, below average wind and swell meant favourable conditions for making sightings, but below average SST was unfavourable). Of course, conditions also vary within each survey year, such that a given variable, say haze, may be below average on one day of the survey and above average on another (thus, acting in different directions when standardizing the observed data to average conditions).

### Biomass per sighting (BpS) model

Observers whose school size estimates could be inter-calibrated showed good consistency (Table 3), with the exception of observer 3, who tended to underestimate school sizes by about 20% relative to the reference observer, and to a lesser extent observer 11, who tended to overestimate school sizes by about 10%.

**Table 3:** Estimated relative observer effects (*β*) and standard error (SE) estimates for the school size calibration model, using observer 5 as the reference level (*β*_5_ *≡* 1).

Observer	*β*	SE(*β*)
3	0.81	0.010
4	1.00	0.012
5	1.00	-
7	0.97	0.011
8	0.95	0.019
11	1.10	0.021

In the BpS model with all environmental covariates, SST was the only one found to be significant (p<0.01) (Table 4). Thus, we refit the model with only SST. The model with only SST explained 45.9% of the deviance (compared to 46.1% with all environmental covariates), and had a much lower AIC value (3.2 units lower with four less parameters), so only SST was retained in the final model. The coefficient for SST is positive (Table 4), indicating that the average biomass of a sighting tends to increase as SST increases, as can be seen from the raw data (Fig 3). The diagnostic tests and plots (provided in S3 Appendix) suggest that the model fits the data well.

**Table 4:** Estimated coefficients for the environmental covariates in the biomass per sighting (BpS) and sightings per mile (SpM) models, with t-tests of significance.

Model	Covariate	Estimate	SE	t-value	p-value
BpS:all	SST	0.311	0.054	5.746	0.000
	Wind speed	-0.019	0.027	-0.721	0.471
	Haze	-0.017	0.063	-0.267	0.789
	Swell	-0.071	0.075	-0.939	0.348
	Sea shadow	0.004	0.020	0.214	0.831
BpS:SST	SST	0.326	0.054	5.999	0.000
SpM	SST	0.328	0.052	6.27	0.000
	Wind speed	-0.305	0.029	-10.67	0.000
	Haze	-0.137	0.061	-2.24	0.025
	Swell	-0.193	0.069	-2.78	0.006
	Sea shadow	-0.053	0.021	-2.59	0.010

For the BpS model, results are presented for the model including all candidate environmental covariates (BpS:all) and the model including only SST (BpS:SST).

### Sightings per mile (SpM) model

Results from the pair-wise observer analysis suggest that observer pairs ranged in their relative sighting efficiency from 63% to 96% compared to the pair with the highest value (Table 5). The pairs that included a trainee spotter (1, 2, 6 or 9) had the lowest estimates.

**Table 5:** Estimated sighting efficiency of each observer pair, relative to the observer pair estimated to have the highest efficiency (5, 11).

Pilot	Spotter	Relative sighting power
4	1	0.76
10	2	0.80
10	1	0.75
4	2	0.81
5	7	0.87
5	3	0.89
4	3	0.86
4	7	0.85
10	6	0.68
10	9	0.63
4	9	0.63
4	6	0.68
5	8	0.88
8	4	0.86
5	4	0.93
8	7	0.82
4	11	0.96
5	11	1.00

All of environmental covariates were found to be significant (p<0.01) in the SpM model (Table 4). The variable with the least support was haze, so we fit a model leaving out haze but it gave a larger AIC value (~3 units higher). Thus, all of the environmental variables were retained in the final SpM model. Based on the coefficient estimates, there is a tendency for the sightings rate to increase as SST increases, and to decline as wind speed, haze, swell and sea shadow increase (Table 4). These relationships are consistent with those seen in the raw data (Fig 3). The coefficients for SST and wind speed are largest in absolute value, indicating that these two variables have the most influence on the sightings rate. The SpM model with all environmental covariates explained 58% of the deviance. The overdispersion (*φ*) and the power (*p*) parameters of the Tweedie distribution were estimated to be 1.78 and 1.13 respectively. The diagnostic tests and plots (provided in S3 Appendix) suggest the model fits quite well. The dispersion test shows a small degree of under-dispersion, suggesting the Tweedie model may be over-fitting, but it is a significant improvement over the Poisson model, and the residual plots show no patterns of concern.

### Relative abundance index

**Fig 7:**
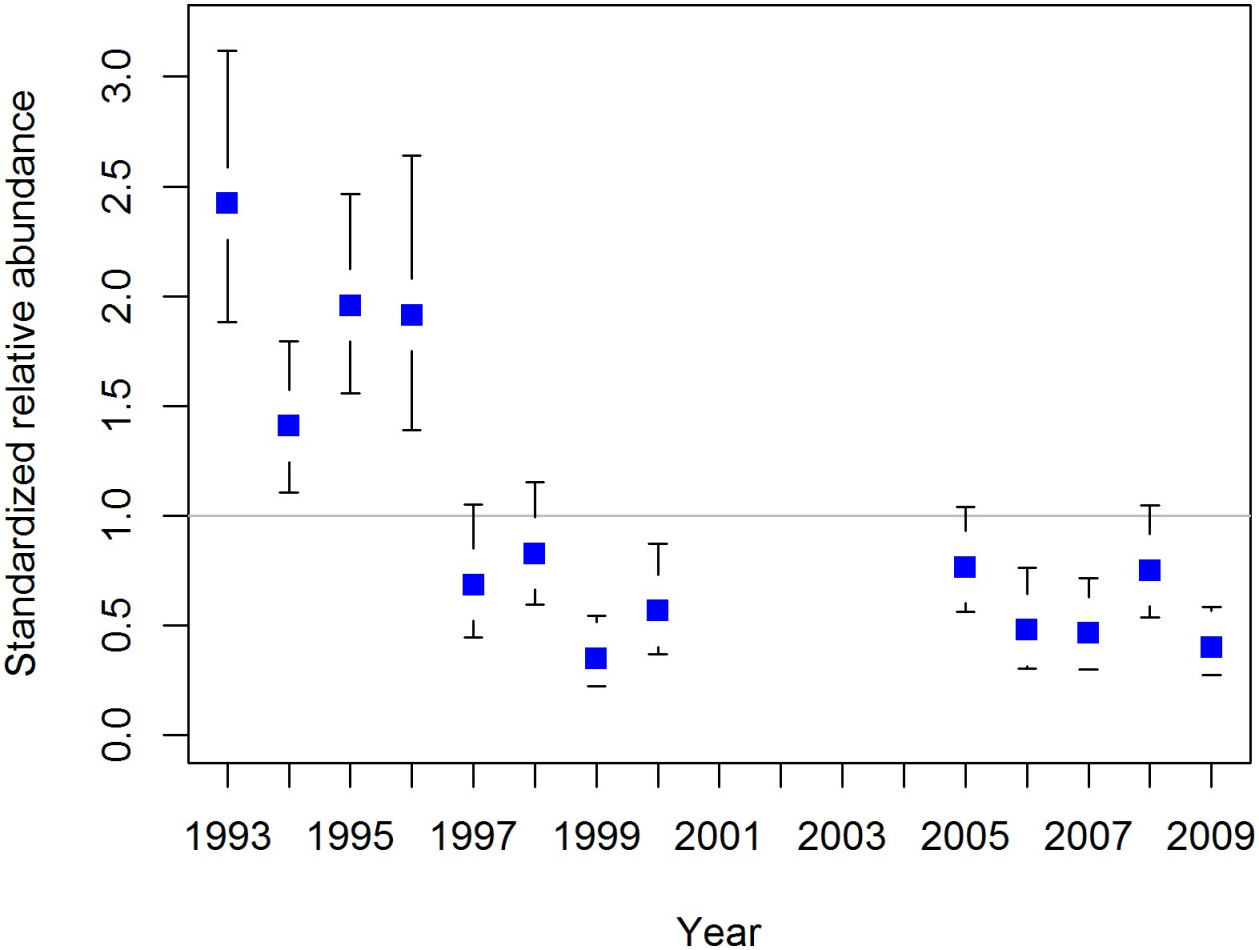
Standardized annual abundance estimates of southern bluefin tuna in the Great Australian Bight, with 95% confidence intervals.

Predicted values from the BpS and SpM models assuming average environmental conditions and the “best” observer pair were used to estimate standardized relative abundance of SBT in the GAB each year. Abundance appears to have been highest in the first years of the survey (1993–1996), followed by a decline in 1997 (Fig 7). From 1997 onwards (noting the missing years between 2001 and 2004) the point estimates fluctuate around a constant level, and the 95% confidence intervals suggest they are not significantly different from each other (Fig 7).

## Discussion

While some aspects of the methods presented are specific to the SBT aerial survey analysis, such as the estimation of observer pair effects for the sightings model, other aspects are more widely applicable. For instance, the use of random effects to model space and time factors allows the model to effectively deal with an imbalanced survey design and could be applicable to line transect data for a number of other fish and marine mammal population—even with the best intentions, there will often be some space and time strata with little or no search effort. Moreover, for species that form schools, such as many fish and dolphins (for which line transect surveys are common), the quantity of interest is not usually the number of sightings but the total number or biomass of animals. In such cases, a method such as the one presented here that models the total number or biomass as two components (the number of sightings and the size/biomass of sightings) may be most appropriate. A similar two-step approach has been used by [20] to estimate dolphin abundance, but bootstrapping was used to estimate variance—the method presented here to estimate variance is more efficient than bootstrapping when random effects are included in the models since each model fit can be quite time-consuming.

We chose to analyse the SBT aerial survey data using a model-based approach as opposed to the more common design-based approach for two main reasons. First, even though the survey design is balanced in space and time, there are many space and time strata for which data could not be collected due to poor weather. Second, because environmental conditions can differ significantly from year to year (e.g. one survey year may be much warmer or windier than another on average), and observers also vary between years, it was important to use a method that standardises for these effects. While there are many benefits of using a model-based approach, perhaps the biggest challenge is model specification—it is not always obvious which covariates to include in the models and the results can be sensitive to the choice. For example, leaving out SST from the SpM model would have led to quite different results. A related question is how much flexibility to allow in the relationship between each covariate and the response variable. Here we assumed linear relationships for all environmental covariates, but this may not always be adequate. The relative advantages of using a design-based, model-based or hybrid approach will depend on the situation, such as whether environmental factors affect animal density and detectability, and whether a formal predefined survey design can be achieved (e.g. [21]).

A significant positive correlation was found between SST and the number of sightings made per mile searched. One reason could be that more fish are at the surface when it is warm; however a study of surfacing behaviour of juvenile SBT in the GAB found SST to have only a minor influence on the proportion of time fish spent at the surface during the day [22]. Another study on the habitat preferences of juvenile SBT in the GAB found SST correlated significantly with where fish tended to be found within the GAB [23]. This suggests that more fish may be spotted in areas that have warmer surface waters because they are more likely to move into these areas. In any case, we would want to account for the influence of SST in the same way in our analysis, since the aim is to adjust the observed number of sightings to reflect how many sightings would have been made if SST was at a standardized level.

The aerial survey analysis has developed over many years as statistical techniques have improved (e.g., software for fitting GLMM models) and new data sets have become available (e.g., fine-scale satellite SST data for the GAB). One area of the analysis that remains unsatisfactory is that the relative sighting power estimates for the various observer pairs get included in the sightings (SpM) model as a known offset, when we know these estimates contain much uncertainty. A goal for future is to formally merge the pair-wise observer model with the SpM model to correctly propagate the uncertainty into the final estimates. General methods for incorporating uncertainty in offsets have been developed in the field of distance sampling [24], but have proven challenging to adapt and implement for our situation.

In interpreting the aerial survey results as an index of juvenile SBT abundance it is important to consider that the index only represents the component of the juvenile SBT population that is in the GAB in any given summer (Jan-Mar). The proportion of the global population of each age class found in the GAB during the summer months is not known, but it is thought to be close to one for ages 2, 3 and 4. This is based largely on data from a large-scale archival tagging project on juvenile SBT, which showed almost 100% of recaptured tagged juveniles of ages 2 to 4 returning to the GAB in summer [12]. The data also showed that most juveniles arrive in the GAB before the start of the survey, and that almost all individuals remain in the survey area until after the survey has ended [12]. Even if the proportion of juveniles in the GAB each summer was not one, as long as the proportion remained similar each year, the data would still provide a valid time series of relative abundance.

The fishery-independent aerial survey for SBT should be distinguished from commercial spotting operations that occur each year in the GAB, in which aerial spotters help commercial purse seine vessels locate fish. Although the commercial spotting data can potentially be used to construct a time-series index of relative abundance [19], they are less reliable than survey data because they are not collected using a pre-set survey design, so spatial and temporal coverage can be limited and inconsistent between years. Moreover, data may not be collected on the full suite of environmental variables required for standardisation, and operational changes in catching operations (e.g. due to changes in quota level or economics) can affect the way that commercial spotting is conducted.

Finally, even though the aerial survey for SBT has continued until 2017, the results presented here only include data to 2009 for reasons explained in the Methods section. We note, however, that estimates from more recent years, derived using modified methods that take into account planes having only one spotter, suggest that the abundance of juveniles in the GAB has increased since 2009 to levels much higher than those observed in the 1993 to 1996 period [25]. Because the aerial survey estimates are a key input to the management procedure used for determining the global quota for SBT, this increasing trend contributed to an increase in the quota for the period 2015–2017 [26].

## Supporting information

S1 Appendix Direct calibration of observer pairs for SpM model. (DOC)

S2 Appendix Calculating variance-covariance matrix of abundance estimates. (DOC)

S3 Appendix Model diagnostics. (DOCX)

S1 Dataset Data required for BpS observer model. (CSV)

S2 Dataset Data required for BpS GLMM. (CSV)

S3 Dataset Data required for SpM observer model. (CSV)

S4 Dataset Data required for SpM GLMM. (CSV)

S1 Code R functions needed to fit models and estimate abundance index. (R)

S2 Code R code to run the analysis. (R)
