# Supplementary material for: Accounting for environmental and observer effects in estimating abundance of southern bluefin tuna from aerial survey data
Source: PLoS One. 2018 Nov 26;13(11):e0207790. doi: 10.1371/journal.pone.0207790 (PMC6257917; doi:10.1371/journal.pone.0207790)
Supplement: S1 Appendix — (DOC) [file pone.0207790.s001.doc]

S1 Appendix. Estimating observer effects for the BpS and SpM models

## Estimating relative observer differences in biomass estimates for the BpS model

Let $b_{t}$ be the true biomass of school *t*, and $B_{t,i}$ be the biomass estimate made by observer *i*. Then

$$B_{t,i}=b_{t}\beta_{i}\varepsilon_{t,i}$$

where $\beta_{i}$ is the observer effect (i.e., the multiplicative factor by which observer *i* tends to over- or under-estimate school size) and $\varepsilon_{t,i}$ is random error.

If observer *j* also estimates the biomass of school *t*, then we have

$$\log\left(\frac{B_{t,i}}{B_{t,j}}\right)=\log\beta_{i}-\log\beta_{j}+\eta_{t}$$

where $\eta_{t}=\log\varepsilon_{t,i}-\log\varepsilon_{t,j}$ and all $\eta_{t}$ are independent. If one observer is chosen as the reference with $\log\beta\equiv0$ (i.e.,$\beta\equiv1$), then a linear model can be constructed with an appropriate design matrix of 1’s, −1’s and 0’s to explain the mean log-differences in biomass estimates across all schools and pairs of observers. Estimates of the observer effects ($\beta$’s) can be obtained by fitting the model using a generalized linear model (we assumed a Gaussian error structure) and taking the exponential of the resulting $\log\beta$ parameter estimates.

We chose observer 5 as the reference observer, since he had the greatest amount of data. Since the trainee observers in 1998 and 1999 (1, 2, 6 and 9) did not make biomass estimates, it was not possible, or necessary, to include them in the calibration. However, the spotting pilot (10) who flew with them, and only them, could not be included either since he did not have overlapping estimates with any other observer. Observers who could be inter-calibrated showed good consistency in their school size estimates, except for observer 3, who tended to underestimate school sizes by about 20% relative to the reference observer, and to a lesser extent observer 11, who tended to overestimate school sizes by about 10% (Table 3 of manuscript). For observer 10 we simply assumed an average calibration value of 1.0. The calibration coefficients were estimated with very high precision, with standard errors ranging from 0.01 to 0.02 (Table 3 of manuscript).

## Estimating relative sighting efficiency of observer pairs for the SpM model

Because spotters operate in pairs (two per plane), it is possible to cross-calibrate individual observers by comparing sighting rates *within* single flights, before having to take into account patterns of sighting rates across space, time, and weather. The rationale is that abundance and sighting conditions are roughly the same for both observers within a flight, and since a flight interchanges flying southward and northward along transect lines, any side-specific differences (e.g. sun angle) are presumed to cancel out on average. Any within-flight differences between the number of sightings by the two observers are attributable to differences in observer sighting efficiency (which is what we want to know), plus statistical noise (which we can adjust for).

The standard protocol for double-observer line-transect surveys is for each observer to remain unaware of what the other has seen. After the survey has finished, each school can be examined to determine whether observer A, observer B, or both observers saw it. This permits a full mark-recapture analysis, in which the probability of both observers missing a school can be inferred based on how many schools were seen by only one observer compared to how many were seen by both. However, in the cramped confines of the planes in the SBT aerial survey, this strict independence protocol is impossible, so other methods of analysis must be developed.

The SBT survey protocol is for each observer to concentrate on his own side of the aircraft, although some sightings (about 17% overall) are “poached”, i.e. made on the opposite side. (Note that “poaching” is not meant pejoratively; the term is borrowed from other line transect work.) A statistical model to describe these observer effects is as follows. Suppose observers *i* and *j* fly as a pair. Let *X* be the time until observer *i* spots a potential sighting on his side of the plane (from the time that the sighting became available for spotting). We assume *X* is exponentially distributed with parameter$\alpha_{i}$ (observer *i*’s instantaneous sightings rate or “sighting efficiency”). Then, the probability observer *i* spots a potential sighting on his side of the plane within the time that it is available for spotting (e.g., from the time it surfaced until it went under) is $1-\exp\left(-\alpha_{i}\right)$. Similarly, let *Y* be the time until observer *i* spots a potential sighting on observer *j*’s side of the plane (from the time that the sighting became available). *Y* is also exponentially distributed, but with parameter $\theta_{i}\alpha_{i}$, where $\theta_{i}$ is observer *i*’s poaching rate. Then, the probability observer *i* spots a potential sighting on observer *j*’s side of the plane within the time that it is available for spotting is $1-\exp\left(-\theta_{i}\alpha_{i}\right)$.

Under the assumption that *X* and *Y* are independent, it can easily be shown that the probability a potential sighting is spotted on *i*’s side of the plane (by either observer *i* or *j*) is $1-\exp\left\{-\left(\alpha_{i}+\theta_{j}\alpha_{j}\right)\right\}$. Also, given that a sighting is made on *i*’s side of the plane, the probability it was made by observer *i* is $\alpha_{i}/\left(\alpha_{i}+\theta_{j}\alpha_{j}\right)$. Thus, the probability that a potential sighting is made on *i*’s side of the plane and it is made by observer *i* is

$p_{ii}=\frac{\alpha_{i}}{\alpha_{i}+\theta_{j}\alpha_{j}}\left(1-\exp\left\{-\left(\alpha_{i}+\theta_{j}\alpha_{j}\right)\right\}\right)$.

Likewise, the probability that a potential sighting is made on *i*’s side of the plane and it is made by observer *j* is

$p_{ij}=\frac{\theta_{j}\alpha_{j}}{\alpha_{i}+\theta_{j}\alpha_{j}}\left(1-\exp\left\{-\left(\alpha_{i}+\theta_{j}\alpha_{j}\right)\right\}\right)$.

Equivalent probability statements,$p_{jj}$ and $p_{ji}$, can be made for sightings on *j*’s side of the plane.

A binomial likelihood for observer pair (*i*, *j*) based on the above probabilities is

$L_{(i,j)}=p_{ii}^{n_{ii}}p_{ij}^{n_{ij}}p_{jj}^{n_{jj}}p_{ji}^{n_{ji}}$,

where $n_{ii}$ is the number of sightings made on *i*’s side of the plane by observer *i*, $n_{ij}$ is the number of sightings made on *i*’s side of the plane by observer *j*, etc. The likelihood over all observer pairs is simply

$L=\prod_{(i,j)} L_{(i,j)}$.

The $\alpha$ and $\theta$ parameters can be estimated by maximizing the overall likelihood. Note that a minimum of three observers, i.e., flying in pairs (*i*, *j*), (*i*, *k*) and (*j*, *k*), is needed for all parameters to be estimable.

The data for all observer pairs that have flown in the SBT aerial surveys from 1993-2009 are given in S1 Table 1. For sightings made very close to the transect line it is not always clear whether they should be considered “own-side” or “poached”; thus, the numbers in S1 Table 1 omit any ambiguous sightings.

The maximum likelihood estimates of the sighting efficiencies ($\alpha$) and poaching rates ($\theta$) are given in S1 Table 2. The experienced observers have reasonably high sighting efficiency estimates, with the probability of making a sighting on their own side of the plane ranging from 0.59 to 1.0 (S1 Table 2). The trainee observers tend to have lower efficiencies, with an own-side sighting probability as low as 0.16 for one of them. There are large differences in the extent of poaching, even amongst the experienced observers. It is interesting to note that the probability of making a poached sighting is zero or close to zero for the trainee observers, and relatively high (0.29 and 0.37) for the experienced observers (4 and 10) who flew with them. The expected numbers of “own-side” and poached sightings calculated for each observer pair using their $\alpha$ and $\theta$ estimates indicate the fit to the data is reasonably good (S1 Table 1).

To adjust for observer pair effects in the SpM model, we need an estimate of the combined sighting power of observers *i* and *j* flying together (i.e., the probability that they will not miss an available sighting). A potential sighting should be equally likely to occur on either side, so the combined sighting power is

$\frac{1}{2}\left(1-\exp\left\{-\left(\alpha_{i}+\theta_{j}\alpha_{j}\right)\right\}\right)+\frac{1}{2}\left(1-\exp\left\{-\left(\alpha_{j}+\theta_{i}\alpha_{i}\right)\right\}\right)$.

The combined sighting power estimates range from 0.70 to 0.97. The ability of the model to resolve estimates of absolute sighting power from the data is weak, but the relative sighting power of one observer pair to another should be reasonably well estimated. As such, we calculated the relative sighting power of every pair compared to the pair with the highest power and carried these values forward into the SpM model (Table 5 of manuscript).

**S1 Table 1. Observed (*n*) and fitted ($\hat{n}$) values for number of “own-side” sightings and “poached” sightings made by all observer pairs (*i, j*) that have flown in the SBT aerial survey.**

| Obs *i* | Obs *j* | # Flights | $n_{ii}$ | $\hat{n}_{ii}$ |  | $n_{ij}$ | $\hat{n}_{ij}$ |  | $n_{ji}$ | $\hat{n}_{ji}$ |  | $n_{jj}$ | $\hat{n}_{jj}$ |
| --- | --- | --- | --- | --- | --- | --- | --- | --- | --- | --- | --- | --- | --- |
| 4 | 3 | 40 | 51 | 50 |  | 9 | 6 |  | 11 | 12 |  | 40 | 43 |
| 5 | 3 | 58 | 137 | 118 |  | 10 | 11 |  | 12 | 23 |  | 85 | 92 |
| 5 | 4 | 40 | 72 | 66 |  | 20 | 12 |  | 14 | 12 |  | 44 | 60 |
| 8 | 4 | 19 | 27 | 25 |  | 10 | 8 |  | 9 | 7 |  | 23 | 29 |
| 4 | 6 | 7 | 11 | 9 |  | 0 | 0 |  | 1 | 3 |  | 2 | 3 |
| 10 | 6 | 6 | 5 | 5 |  | 0 | 0 |  | 2 | 2 |  | 2 | 2 |
| 4 | 7 | 17 | 33 | 30 |  | 10 | 13 |  | 6 | 10 |  | 28 | 25 |
| 5 | 7 | 61 | 101 | 107 |  | 33 | 34 |  | 35 | 27 |  | 80 | 80 |
| 8 | 7 | 11 | 17 | 15 |  | 13 | 9 |  | 3 | 6 |  | 12 | 15 |
| 5 | 8 | 2 | 7 | 6 |  | 1 | 1 |  | 1 | 1 |  | 4 | 5 |
| 4 | 9 | 5 | 10 | 9 |  | 0 | 0 |  | 3 | 3 |  | 0 | 2 |
| 10 | 9 | 5 | 8 | 8 |  | 0 | 0 |  | 3 | 4 |  | 3 | 2 |
| 4 | 1 | 6 | 22 | 21 |  | 1 | 1 |  | 2 | 6 |  | 14 | 11 |
| 10 | 1 | 5 | 8 | 6 |  | 0 | 0 |  | 4 | 2 |  | 0 | 3 |
| 4 | 2 | 7 | 8 | 5 |  | 0 | 0 |  | 1 | 1 |  | 2 | 4 |
| 10 | 2 | 7 | 5 | 6 |  | 1 | 1 |  | 2 | 2 |  | 6 | 5 |
| 4 | 11 | 39 | 73 | 57 |  | 4 | 4 |  | 3 | 3 |  | 56 | 73 |
| 5 | 11 | 15 | 36 | 46 |  | 2 | 2 |  | 1 | 2 |  | 65 | 54 |

**S1 Table 2. Estimated “own-side” sighting rate ($\alpha$) and “opposite side” poaching rate ($\theta$) of each observer, plus the probability of each observer making a potential sighting on his own side ($1-e^{-\alpha}$) and on the opposite side ($1-e^{-\theta\alpha}$).**

| Observer | $\alpha$ | $\theta$ | $1-e^{-\alpha}$ | $1-e^{-\theta\alpha}$ |
| --- | --- | --- | --- | --- |
| 1* | 0.67 | 0.07 | 0.49 | 0.05 |
| 2* | 0.93 | 0.12 | 0.60 | 0.10 |
| 3 | 1.25 | 0.15 | 0.71 | 0.17 |
| 4^ | 1.50 | 0.23 | 0.78 | 0.29 |
| 5^ | 1.97 | 0.16 | 0.86 | 0.27 |
| 6* | 0.34 | 0.00 | 0.29 | 0.00 |
| 7 | 0.90 | 0.71 | 0.59 | 0.47 |
| 8^ | 1.07 | 0.34 | 0.66 | 0.31 |
| 9* | 0.18 | 0.00 | 0.16 | 0.00 |
| 10^ | 1.25 | 0.38 | 0.71 | 0.37 |
| 11 | 9.95 | 0.01 | 1.00 | 0.09 |

* Indicates a trainee observer

^ Indicates an observer also operating as the pilot (referred to as a “spotting pilot” in the main text).
