# Supplementary material for: Accounting for environmental and observer effects in estimating abundance of southern bluefin tuna from aerial survey data
Source: PLoS One. 2018 Nov 26;13(11):e0207790. doi: 10.1371/journal.pone.0207790 (PMC6257917; doi:10.1371/journal.pone.0207790)
Supplement: S2 Appendix — (DOC) [file pone.0207790.s002.doc]

# S2 Appendix. Calculating the variance-covariance matrix of the annual relative abundance estimates

Let $\hat{B}_{ijk}$ be the predicted value of BpS in year *i*, month *j* and area *k* under standardized environmental/observer conditions, and $\hat{\sigma}\left(\hat{B}_{ijk}\right)$ be its estimated standard error. Similarly, let $\hat{S}_{ijk}$ be the predicted value of SpM in year *i*, month *j* and area *k* under the same environmental/observer conditions, and $\hat{\sigma}\left(\hat{S}_{ijk}\right)$ be its estimated standard error. Then,

$$\hat{A}_{ijk}=\hat{S}_{ijk}\hat{B}_{ijk}$$

is the stratum-specific abundance estimate for year *i*, month *j* and area *k*.

Since$\hat{B}_{ijk}$ and $\hat{S}_{ijk}$ are independent, the variance of $\hat{A}_{ijk}$ is given by

$$\begin{matrix} V\left(\hat{A}_{ijk}\right)=V\left(\hat{S}_{ijk}\hat{B}_{ijk}\right) \\ =V\left(\hat{S}_{ijk}\right)E\left(\hat{B}_{ijk}\right)^{2}+V\left(\hat{B}_{ijk}\right)E\left(\hat{S}_{ijk}\right)^{2}+V\left(\hat{S}_{ijk}\right)V\left(\hat{B}_{ijk}\right) \\ \approx\hat{\sigma}^{2} \left(\hat{S}_{ijk}\right)\hat{B}_{ijk}^{2}+\hat{\sigma}^{2} \left(\hat{B}_{ijk}\right)\hat{S}_{ijk}^{2}+\hat{\sigma}^{2} \left(\hat{S}_{ijk}\right)\hat{\sigma}^{2} \left(\hat{B}_{ijk}\right) \end{matrix}$$

The annual abundance estimate for year *i* is given by the weighted sum of all stratum-specific abundance estimates within the year, namely

$$\hat{A}_{ i}=\sum_{j} \sum_{k} w_{k}\hat{A}_{ijk}$$

where $w_{k}$ is the size of area *k* relative to the entire survey area ($\sum_{k} w_{k} = 1$).

If the $\hat{A}_{ijk}$’s are independent, then the variance of $\hat{A}_{i}$ is given by

$$V\left(\hat{A}_{i}\right)=\sum_{j} \sum_{k} w_{k}^{2} V\left(\hat{A}_{ijk}\right)$$

Unfortunately, the $\hat{A}_{ijk}$’s are NOT independent because the estimates of BpS (and likewise, the estimates of SpM) are not independent between different strata. This is because all strata estimates depend on the estimated coefficients of the environmental/observer conditions, so any error in these estimated coefficients will affect all strata. Thus, we refit the BpS and SpM models with the coefficients of the environmental/observer covariates (denote the vector of coefficients by $\theta$) fixed at their estimated values ($\hat{\theta}$). Note that $\theta$ contains the environmental/observer coefficients from both the BpS and SpM models; i.e. $\theta=(\theta_{\text{BpS}},\theta_{\text{SpM}})$. The predictions of BpS and SpM made using the ‘fixed environment’ models should now be independent between strata, so the stratum-specific abundance estimates calculated using these predictions – which we will denote by $\hat{A}_{ijk}\left(\hat{\theta}\right)$ – should also be independent between strata. Thus, we can calculate the variance of $\hat{A}_{i}$ conditional on the estimated values of the environmental/observer coefficients as

$$V\left(\hat{A}_{i}|\hat{\theta}\right)=\sum_{j} \sum_{k} w_{k}^{2} V\left(\hat{A}_{ijk}\left(\hat{\theta}\right)\right)$$

where $V\left(\hat{A}_{ijk}\left(\hat{\theta}\right)\right)$ is calculated using the formula given above for $V\left(\hat{A}_{ijk}\right)$ but using the BpS and SpM predictions and standard errors obtained from the ‘fixed environment’ models.

To calculate the unconditional variance of $\hat{A}_{i}$, we make use of the following equation:

$$\begin{matrix} V\left(\hat{A}_{i}\right)=E_{\theta}\left(V\left(\hat{A}_{i}|\theta\right)\right)+V_{\theta}\left(E\left(\hat{A}_{i}|\theta\right)\right) \\ \approx V\left(\hat{A}_{i}|\hat{\theta}\right)+V_{\theta}\left(\hat{A}_{i}\right) \end{matrix}$$

where the first term is the conditional variance just discussed and the second term is the additional variance due to uncertainty in the environmental coefficients. The second term can be estimated as follows

$$V_{\theta}\left(\hat{A}_{i}\right)\approx\left(\frac{\partial\hat{A}_{i}}{\partial\theta}\right)^{T}\mathbf{V}_{\theta}\left(\frac{\partial\hat{A}_{i}}{\partial\theta}\right)$$

where $\left(\frac{\partial\hat{A}_{i}}{\partial\theta}\right)$ is the vector of partial derivatives of $\hat{A}_{i}$ with respect to $\theta$ (which we calculated using numerical differentiation), and $\mathbf{V}_{\theta}$ is the variance-covariance matrix of the environmental coefficients. Recall that $\theta$ contains the environmental coefficients from both the BpS and SpM models, so $\mathbf{V}_{\theta}=\left[\begin{matrix} \mathbf{V}_{\theta_{\text{BpS}}} & \mathbf{0} \\ \mathbf{0} & \mathbf{V}_{\theta_{\text{SpM}}} \end{matrix}\right]$, where the variance-covariance matrices for the individual models are returned from the model-fitting software.
