# Supplementary material for: Accounting for environmental and observer effects in estimating abundance of southern bluefin tuna from aerial survey data
Source: PLoS One. 2018 Nov 26;13(11):e0207790. doi: 10.1371/journal.pone.0207790 (PMC6257917; doi:10.1371/journal.pone.0207790)
Supplement: S3 Appendix — (DOCX) [file pone.0207790.s003.docx]

# S3 Appendix. Model diagnostics

Diagnostic tools provided by the DHARMa package in R (Hartig 2018) were used to evaluate the model fits. DHARMa simulates quantile residuals from a fitted GLMM that are standardized to values between 0 and 1. For a correctly specified model, these residuals should have a uniform distribution regardless of the underlying model structure, and can be interpreted similarly to residuals for linear models. The package includes statistical tests on the residuals to check for uniformity, overdispersion and zero inflation. There is also a function to plot the residuals against covariates in the model (or potential covariates not in the model) to look for possible misspecifications; for a correctly specified model, the residuals should be uniform in the y direction (i.e. flat with no systematic pattern with the covariates). Details can be found in the reference manual and vignette available from <https://cran.r-project.org/web/packages/DHARMa/index.html>.

Note that one difficulty we encountered with using DHARMa for the SpM model is that the functions to simulate residuals have not yet been implemented for the Tweedie distribution for models fit using gam – but they have (very recently) for models fit using glmmTMB (Brooks et al. 2017). Thus, for diagnostic checking, we fit the SpM model using the glmmTMB function (noting that the coefficient estimates obtained using gam and glmmTMB had a correlation >0.999).

**Biomass per sighting (BpS) model**

Diagnostic plots for the BpS model suggest that model fits the data well. The Q-Q plot to check for uniformity of the residuals if very close to linear, and the formal test confirms this (S3 Fig 1, left). The dispersion plot and formal test indicates no evidence of over (or under) dispersion (S3 Fig 1, right). There is no need to test for zero inflation in the model since all biomass estimates are positive. Plots of the standardized quantile residuals against the covariates included in the model, as well as the potential environmental covariates not included, are quite flat and show no patterns of concern (S3 Fig 2).

**Sightings per mile (SpM) model**

Diagnostic plots and test for the SpM model fitted assuming a Poisson error structure suggest a significant lack of fit, with overdispersion and zero-inflation being apparent in the residuals (S3 Fig 3). The model fit was much improved when a Tweedie error structure was used (S3 Fig 4). The dispersion test indicates that the model may be slightly overfitting (since the residuals appear to be under-dispersed), but otherwise the tests for uniformity and zero inflation indicate a good fit, and plots of the residuals against the model covariates are quite flat and show no patterns that suggest model misspecification (S3 Fig 5).


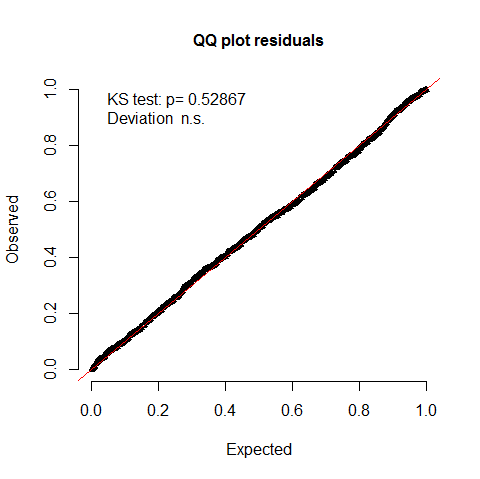


S3 Fig 1. Diagnostic plots and test results for the biomass per sighting (BpS) model. Left: Q-Q plot and test for uniformity; there is no indication of lack of fit (p=0.53). Right: Dispersion test results, showing the histogram of simulated test statistic values compared to the observed value from the fitted model (vertical red line); there is no indication of under- or over-dispersion in the residuals (p=0.24).

S3 Fig 2. Plots of the standardized quantile residuals against the covariates included in the biomass per sighting (BpS) model, as well as the environmental variables not included (wind speed, haze, swell and sea shadow). Horizontal red lines are the 0.25, 0.5 and 0.75 quantile regression lines, to aid visual detection of non-uniformity.

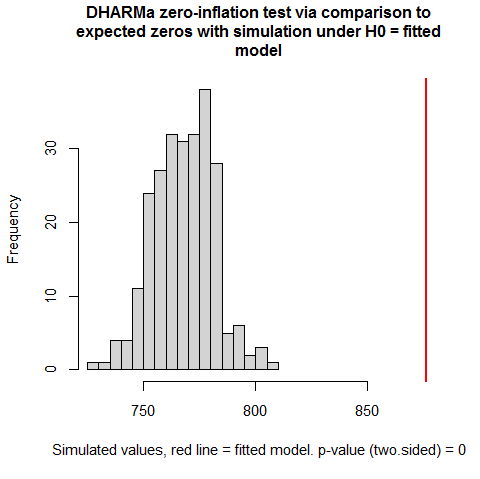


S3 Fig 3. Diagnostics plots and test results for the sightings per mile (SpM) model fitted assuming a Poisson error structure. *Top left:* Q-Q plot and test for uniformity, suggests a significant lack of fit (p<0.001). *Top right:* Dispersion test results, showing the histogram of simulated test statistic values compared to the observed value from the fitted model (vertical red line); there is strong evidence of over-dispersion in the residuals (p<0.001). *Bottom left:* Zero-inflation test results, showing the histogram of simulated test statistic values compared to the observed value from the fitted model (vertical red line); there is strong evidence of zero-inflation in the residuals (p<0.001).

S3 Fig 4. Diagnostics plots and test results for the sightings per mile (SpM) model fitted assuming a Tweedie error structure. *Top left:* Q-Q plot and test for uniformity; there is no indication of lack of fit (p=0.28). *Top right:* Dispersion test results, showing the histogram of simulated test statistic values compared to the observed value from the fitted model (vertical red line); there is evidence of under-dispersion in the residuals (p<0.001). *Bottom left:* Zero-inflation test results, showing the histogram of simulated test statistic values compared to the observed value from the fitted model (vertical red line); there is no evidence of zero-inflation in the residuals (p=0.46).

S3 Fig 5. Plots of the standardized quantile residuals against the covariates included in the sightings per mile (SpM) model fitted assuming a Tweedie error structure. Horizontal red lines are the 0.25, 0.5 and 0.75 quantile regression lines, to aid visual detection of non-uniformity.

**References**

Hartig F. DHARMa: Residual Diagnostics for Hierarchical (Multi-Level / Mixed) Regression Models. R package version 0.2.0. <https://CRAN.R-project.org/package=DHARMa>. 2018.

Brooks ME, Kristensen K, Benthem KJ, Magnusson A, Berg CW, Nielsen A, Skaug HJ, Maechler M, Bolker BM. glmmTMB Balances Speed and Flexibility Among Packages for Zero-inflated Generalized Linear Mixed Modeling. The R Journal. 2017;9(2):378-400.
